# Administration of zoledronic acid alleviates osteoporosis in HIV patients by suppressing osteoclastogenesis via regulating RANKL expression

**DOI:** 10.1186/s10020-021-00276-5

**Published:** 2021-02-26

**Authors:** Wei Lin, Xing-fu Li, Dong-cheng Ren, Meng Song, Li Duan, Jin-zhu Liu, Zi-rui Zhan

**Affiliations:** 1 Department of Orthopedics, Third People’s Hospital of Shenzhen, No. 29 Bulan Road, Longgang, Shenzhen, 518112 Guangdong People’s Republic of China; 2 Department of Orthopedics, Shenzhen No. 2 People’s Hospital, Shenzhen, 518000 People’s Republic of China

**Keywords:** Osteoporosis, Zoledronic acid, HIV, RANKL, miRNA, Osteoclastogenesis

## Abstract

**Background:**

Osteoporosis is a common phenomenon in HIV patients on tenofovir treatment, but its underlying mechanisms remain to be explored.

**Methods:**

Quantitative real-time PCR was performed to analyze the expression of miR-302, miR-101, miR-145 and osteoclast-specific genes in the serum of HIV patients treated with tenofovir and ZOL. ELISA was used to evaluate the expression of RANKL, SMAD3 and PRKACB in the serum of these patients. Luciferase assay was carried out to explore the inhibitory effects of miR-302, miR-101 and miR-145 on the expression of PRKACB, RANKL and SMAD3, respectively. Western blot was used to examine the expression of genes involved in NF‑κB and JNK signaling pathways.

**Results:**

ZOL treatment significantly suppressed the expression of CTx and osteocalcin in HIV patients treated with tenofovir. The BMD loss of HIV patients treated with tenofovir was effectively hindered by ZOL treatment. Mechanistically, the expression of miR-302, miR-101, miR-145, RANKL, SMAD3 and PRKACB in the serum was remarkably activated by ZOL treatment. Luciferase assays showed that miR-302, miR-101 and miR-145 effectively suppressed the expression of PRKACB, RANKL and SMAD3, respectively, through binding to their 3′ UTR. Furthermore, ZOL treatment notably restored the normal expression of osteoclast‑specific genes while activating NF‑κB and JNK signaling pathways.

**Conclusion:**

The findings of this study demonstrated that administration of ZOL suppressed the expression of RANKL via modulating signaling pathways of miR-101-3p/RANKL, miR-302/PRKACB/RANKL and miR-145/SMAD3/RANKL. Furthermore, down-regulated expression of RANKL by ZOL treatment alleviated osteoporosis in HIV-positive subjects treated with tenofovir.

## Introduction

Osteoporosis is a typical problem impacting approximately 20 million senior women as well as 6 million senior males in Europe in 2010, with approximately 1/3 female population as well as 1/5 male population likely to suffer an osteoporotic fracture in their life (Hernlund et al. 2013). Individuals with HIV infection are more likely to have reduced bone mineral density (BMD) as well as osteoporosis (Mallon 2010). Some have actually suggested that HIV be treated as an additional source of osteoporosis, although HIV itself has actually not been thought as a risk factor in the progression of osteoporosis (Brown 2013). Just recently, European standards included the upkeep of bone health as a factor to consider in the selection of antiretroviral treatment for HIV (Malde et al. 2018).

ZOL is among the options in the therapy of ADIS. The favorable impact of ZOL was mainly discovered after the initial dose, increasing the chance that one dose may be adequate for subjects with a reduced BMD. Continual rise in BMD at the hip as well as the lumbar spine has actually been shown in HIV positive males (Bolland et al. 2012; Grey et al. 2017). This would certainly make ZOL a basic as well as reasonably economical choice for enhancing BMD in those people without access for an ART based therapy program (Carr et al. 2019).

MicroRNAs (miRNAs) are conserved tiny and non-coding RNAs of about 22 nucleotides in length. MiRNA plays a vital function in post-transcriptional control of target mRNAs as well as translational inhibition of proteins (Tano et al. (2011)). MiRNAs are found in many types of cells to subdue the activity of target gene 3′-UTRs, thus are involved in various processes including cell apoptosis, expansion, differentiation, as well as metabolism (Bork et al. 2011). It was actually revealed by miRNA microarrays that the miRNA expression is changed throughout osteoclastogenesis. For example, miR-145 is suppressed throughout osteoclastogenesis caused by RANKL. In vitro, the gain of function of miR-145 might reduce osteoclastogenesis via targeting Smad3 (Yu et al. 2018; Irwandi et al. 2018). Higher expression of miR-302a-3p in HMOBs revealed that PGE2 treatment down-regulated PRKACB. MiR-302a-3p transfection subdued RANKL mRNA expression, playing a significant function in causing osteoclastogenesis (Ikeda et al. 2003, 2001). On the contrary, reduction of RANKL expression in cells treated with PGE2-IFNγ was alleviated after miR-302a-3p was reduced by miRNA-302a-3p inhibitor transfection. These data showed the repressive impact of miR-302a-3p on mRNA expression of PRKACB to cause downregulated expression of RANKL in HMOBs (Irwandi et al. 2018). In this study, we selected 3 miRNAs including miR-302, miR-101 and miR-145 since they were previously reported to participate signaling pathways in the pathogenesis of various diseases (Musolino et al. 2018; Fanale et al. 2016a; Zarone et al. 2017).

RAW 264.7 cells might be turned right into osteoclasts by receptor activator of nuclear factor-κB ligand (RANKL), which belongs to the family of tumor necrosis factor (TNF) and is one of the most important receptors for osteoclast activation as well as development (Abe et al. 2017; Mediero et al. 2013). After receptor binding, RANKL boosts the differentiation of monocyte macrophage to osteoclast (Lacey et al. 1998; Yuan et al. 2016). The binding of RANKL to RANK can also recruit TNF receptor-associated factor 6 (TRAF6) while sequentially activating NF-κB as well as numerous pathways of mitogen-activated protein kinase (MAPK) signaling, such as c-Jun N-terminal kinase (JNK), extracellular signal-regulated kinase (ERK) 1/2, as well as p38 signaling pathways (Wada et al. 2006; Huang et al. 2010; Ihn et al. 2015). Amongst the 3 significant subsets of macrophages, i.e., M2, M1, as well as M0 subsets, the repressive impacts on RANKL-induced osteoclastogenesis are seen in M1 macrophages only. Evaluations showed that the suppression of osteoclastogenesis via M1 seemed to be moderated by the synthesis of IL-12 and IFN-γ, which downregulate NFATc1 induction as well as enhancing apoptosis. Nevertheless, the influence of macrophages on RANKL-induced osteoclastogenesis remains vague (Yamaguchi et al. 2016; Guerrini and Takayanagi 2014; Danks and Takayanagi 2013).

In this study, we collected samples from HIV patients treated with or without ZOL to study its effect on the bone density as well as signaling pathways of ZOL/miR-302/PRKACB/RANKL, ZOL/miR-101/RANKL and ZOL/miR-145/SMAD3/RANKL.

## Materials and methods

### Human subjects sample collection

In this study, we recruited a group of HIV positive patients receiving tenofovir treatment who were diagnosed with osteoporosis. The patients were divided into two groups, and one of the two groups received ZOL treatment (ZOL+ , N = 36) while the other group received no ZOL treatment and was used as control (ZOL−, N = 38). The information of participants including their age, sex, race, history of smoking, current smoking status, cigarettes smoked per day, years of cigarette smoking history, history of bone fracture, HIV-1 RNA level, CD4 count, serum calcium level as well as serum citamin D level, was summarized and compared between the two groups. All subjects gave written form of informed consent, which was reviewed and accepted by the Institutional Ethical Committee.

### Objectives

The primary objectives were to review whether ZOL relieves bone resorption induced by ART. The secondary objectives were to review the effects of ZOL on BMD as well as the safety of ZOL.

### Subjects

HIV infected treatment-naive individuals (with an HIV-1 RNA titer of > 1000 copies/mL) with an age of 30 to 50 years who were preparing ART initiation were enrolled for the research if serum level of vitamin D3 was > 12 ng/mL and their serum level of calcium was > 8 mg/dL.

### Randomization

The subjects were stratified and randomized in accordance to HIV-1 RNA titer (< 100,000 copies/mL or ≥ 100,000 copies/mL), age (30 to 39 years old or 40 to 49 years old), as well as sex.

### Treatment

At admission, the subjects started ART per the requirement for standard treatment by using ritonavir + TDF, atazanavir or emtricitabine. Modification to the ART therapy was permitted when virologic failure or drug intolerance occurred. Upon ART initiation, the subjects additionally received an intravenous injection of 0.05 mg/mL of ZOL or an intravenous injection of 2.2 mg/ml mannitol and 0.24 mg/ml sodium citrate as placebo.

### Follow-up

Research results were evaluated at baseline as well as at week 12, week 24, week 36, and week 48. The research was unblinded when the last enrolled subject finished the visit scheduled for 24 weeks after dosing. Safety examinations were done in week 2, week 12, as well as once every 3 months afterwards.

### Outcome measures

Blood specimens were treated in 60 min after collection to isolate plasma via centrifugation. ELISA kits (Immunodiagnostic Systems, Scottsdale, AZ) were made use of according to the guidelines provided by the manufacturer to measure plasma content of C-Terminal Cross-Linking Telopeptide (CTx), osteocalcin, as well as bone resorption markers. BMD was evaluated by making use of a GE scanner (GE Lunar, Madison, WI) using dual energy X-ray absorptiometry. Osteopenia was specified as a t score of − 1.0 to − 2.5, while osteoporosis was specified as a t score of < − 2.5.

### Sample size and power considerations

Pilot results from a research on treatment naive and HIV infected subjects treated with lopinavir or ritonavir + TDF/FTC for 24 weeks (Musolino et al. 2018) were used as the basis for determining sample size. Based on a typical increase of 1.2 µg/L CTx in the placebo group while no changes in the ZOL group as well as an assumed standard deviation of 1.4 µg/L in each group, a sample size of 30 subjects in each group could obtain a 90% power if α = 0.05.

### RNA isolation and real-time PCR

Total RNA content was isolated from cell and tissue samples by utilizing a Trizol reagent (Invitrogen, Carlsbad, CA) according to the guidelines provided by the manufacturer. The RNA samples were examined by 1% agarose gel electrophoresis to confirm their high quality. In the next step, total RNA was subjected to reverse transcription by utilizing a reverse transcription assay kit (Takara, Tokyo, Japan) according to the guidelines provided by the manufacturer. Then, real time PCR was done by using an ABI Prism 7900HT real time PCR system (Applied Biosystems, Foster City, CA) in conjunction with a SYBR Pre-mix Ex Taq II assay kit (Takara, Tokyo, Japan) according to the guidelines provided by the manufacturer. The relative expression of miR-302, miR-101-3p, miR-145, CTR mRNA, DC-STAMP, RANK, CATCH, c-fos mRNA, and NFATc1 mRNA was evaluated by utilizing the 2^−ΔΔCt^ approach. U6 and GAPDH were respectively used as reference genes for the quantification of miRNA and mRNAs.

### Cell culture and transfection

Human osteoclast precursor cells were maintained in MEM added with appropriate antibiotics, i.e., 100 µg/ml streptomycin and 100 U/ml of penicillin bought LONZA, GE, along with 10% FBS. Culture was done at 37 ˚C in a humidified carbon dioxide incubator. Then, the cells were divided into 3 groups, i.e., (1) NC group (untreated human osteoclast precursor cells); (2) RANKL group (human osteoclast precursor cells treated with RANKL); (3) RANKL + ZOL + Scramble control group (human osteoclast precursor cells treated with both RANKL, ZOL and miRNA inhibitor scramble controls); and (4) RANKL + ZOL + miRNA inhibitors (human osteoclast precursor cells treated with both RANKL, ZOL and miRNA inhibitors including miR-302 inhibitors, miR-101 inhibitors and miR-145 inhibitors). In the RANKL group, human osteoclast precursor cells were treated with 100 ng/ml of RANKL. In the RANKL + ZOL + Scramble control or RANKL + ZOL + miRNA inhibitors group, human osteoclast precursor cells were treated with 100 ng/ml of RANKL as well as 50 µM of ZOL. The treatment lasted for 5 days, and relevant indicators were observed daily.

### Vector construction, mutagenesis and luciferase assay

Our binding target screening of miR-302, miR-101 as well as miR-145 indicated that miR-302 could bind to the 3′ UTR of PRKACB, miR-101 could bind to the 3′ UTR of RANKL, while miR-145 could bind to the 3′ UTR of SMAD3. To further confirm the regulatory roles of miR-302, miR-101 as well as miR-145 in the expression of PRKACB, RANKL, and SMAD3, the 3′ UTRs of PRKACB, RANKL, and SMAD3 containing the binding sites for their respective targeting miRs were inserted into pGL3 vectors (Promega, Madison, WI) to generate wild type vectors for 3′ UTRs of PRKACB, RANKL, and SMAD3. Then, a Quick Change XL Site Directed Mutagenesis assay kit (Stratagene, La Jolla, CA) was used according to the guidelines provided by the manufacturer to induce site directed mutagenesis and to generate mutant type vectors for 3′ UTRs of PRKACB, RANKL, and SMAD3. In the next step, human osteoclast precursor cells were co-transfected with wild type or mutant type vectors of 3′ UTRs of PRKACB, RANKL, and SMAD3 in conjunction with miR-302, miR-101-3p or miR-145, respectively. The transfection was carried out by utilizing Lipofectamine 2000 (Invitrogen, Carlsbad, CA) according to the guidelines provided by the manufacturer. The luciferase assay was done at 48 h after the start of transfection by utilizing a Dual Luciferase Reporter Gene Assay System (Promega, Madison, MI) according to the guidelines provided by the manufacturer.

### Cell proliferation assay

The effect of different ZOL concentrations on the viability as well as growth of human osteoclast precursor cells cultured with or without RANKL was tested by utilizing a CCK-8 assay kit (Thermo Fisher Scientific, Waltham, MA) according to the guidelines provided by the manufacturer. The optical density of treated cells was read at the wavelength of 450 nm by using an ELX800 microplate reader (BioTek, Winooski, VT) according to the guidelines provided by the manufacturer.

### Western blot

Total content of protein was collected from cell and tissue samples via lysis in a RIPA buffer (Thermo Fisher Scientific, Waltham, MA) and subsequent centrifugation. Then, equal quantities (40 μg) of protein samples were loaded onto a 10% SDS-PAGE gel and resolved by electrophoresis, blotted onto a PVDF membrane, blocked with 5% milk, and incubated with primary antibodies against p‑IκBα, IκBα, p‑p65, p65, p‑JNK, JNK, p‑p38, p38, p‑ERK and ERK (Abcam, Cambridge, MA). In the next step, the membrane was further incubated with horseradish peroxidase conjugated secondary antibodies (Sigma Aldrich, St. Louis, MO) at for 1 h at ambient temperature and developed with an enhanced chemiluminescence reagent (ECL, Santa Cruz Biotechnology, Santa Cruz, CA) according to the guidelines provided by the manufacturer to evaluate the relative protein expression of p‑IκBα, IκBα, p‑p65, p65, p‑JNK, JNK, p‑p38, p38, p‑ERK and ERK in each sample. The optic density of each protein band was calculated using Analysis Software (Ultra-Violet Products). Each observation was performed in triplicate.

### ELISA

The contents of RANKL, SMAD3, and PRKACB in collected samples were measured by using commercial ELISA kits (Thermo Fisher Scientific, Waltham, MA) according to the guidelines provided by the manufacturer.

### Statistical analysis

All results were shown as mean ± standard deviation. One-way analysis of variance (ANOVA) and Student’s t tests were utilized to compare inter-group differences. Statistical analyses were done by making use of SPSS 22.0 software (SPSS, Chicago, IL). P values of < 0.05 were deemed statistically significant.

## Results

### The characteristics of patients

We recruited a group of HIV positive patients receiving tenofovir treatment who were diagnosed with osteoporosis. The patients were divided into two groups, and one of the two groups received ZOL treatment (ZOL+ , N = 36) while the other group received no ZOL treatment and was used as control (ZOL−, N = 38). The characteristics of participants were summarized in Table 1. Student’s t test was utilized to perform statistical comparison, and the results revealed no obvious difference between the two groups.

**Table 1:** Basic characteristics of HIV positive subjects

Characteristics	ZOL(−) (N = 38)	ZOL(+) (N = 36)	*P* value
Age, year	41.2 ± 6.8	40.8 ± 5.6	0.331
Sex, male (%)	31 (81.6)	30 (83.3)	0.324
Race			0.520
White	13 (34.2)	12 (33.3)	
Black	25 (65.8)	24 (66.7)	
History of smoking			0.421
Yes	31 (81.6)	31 (86.1)	
No	7 (18.4)	5 (13.9)	
Current smoking			0.699
Yes	29 (76.3)	30 (83.3)	
No	9 (23.7)	6 (16.7)	
Cigarettes smoked per day (in patients with history of smoking)	3 (7.9)	3 (8.3)	0.383
Years of cigarette smoking (in patients with history of smoking)	3 (7.9)	4 (11.1 + G14:N14)	0.349
History of bone fracture			0.636
Yes	8 (21.1)	5 (13.9)	
No	30 (78.9)	31 (86.1)	
HIV RNA, log^10^ copies/mL (SD)	4.1 ± 0.8	3.7 ± 0.7	0.536
CD4 count, cells/uL (SD)	159.7 ± 37.7	153.3 ± 44.9	0.498
Serum calcium, mg/dL(SD)	9.8 ± 3.8	9.7 ± 2.6	0.568
Serum vitamin D, ng/mL(SD)	24.9 ± 6.1	24.3 ± 5.7	0.430

### ZOL treatment suppressed CTx and Osteocalcin expression in HIV patients receiving tenofovir treatment

**Fig. 1:**
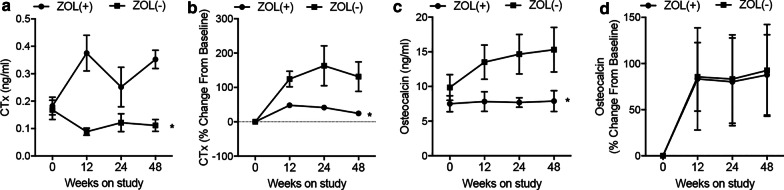
CTx and Osteocalcin expression was suppressed by ZOL treatment in HIV patients receiving tenofovir treatment. **a** Model-based mean longitudinal changes of CTx concentration in 48 weeks in ZOL-treated HIV patients receiving tenofovir treatment. **b** Model-based mean CTx percentage change from baseline in 48 weeks in ZOL-treated HIV patients receiving tenofovir treatment. **c** Model-based mean longitudinal changes of Osteocalcin concentration in 48 weeks in ZOL-treated HIV patients receiving tenofovir treatment. **d** Model-based mean Osteocalcin percentage change from baseline in 48 weeks in ZOL-treated HIV patients receiving tenofovir treatment

CTx and Osteocalcin expression, which was indicative of bone resorption and formation, was measured for 48 weeks. The mean CTx and Osteocalcin concentrations were decreased in the ZOL + group compared with the ZOL− group (Fig. 1a, c). Accordingly, the mean CTx percentage were also lower in the ZOL + group compared with the ZOL− group (Fig. 1b), whereas the mean Osteocalcin percentage in ZOL + group is comparable with that in the ZOL− group (Fig. 1d).

### ZOL treatment prevented BMD Loss in HIV patients receiving tenofovir treatment

**Fig. 2:**
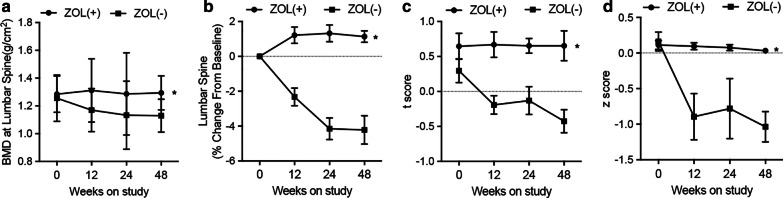
BMD Loss was repressed by ZOL treatment in HIV patients receiving tenofovir treatment. **a** Model-based mean longitudinal changes in BMD at the lumbar spine in 48 weeks in ZOL-treated HIV patients receiving tenofovir treatment. **b** Model-based mean BMD percentage change at the lumbar spine from baseline in 48 weeks in ZOL-treated HIV patients receiving tenofovir treatment. **c** Model-based mean longitudinal changes in lumbar spine t scores in 48 weeks in ZOL-treated HIV patients receiving tenofovir treatment. **d** Model-based mean longitudinal changes in lumbar spine z scores in 48 weeks in ZOL-treated HIV patients receiving tenofovir treatment

Next, the BMD at lumbar spine was measured to evaluate the effect of ZOL on BMD loss. The BMD at lumbar spine in the ZOL + group was significantly increased compared with the ZOL− group (Fig. 2a). Furthermore, ZOL treatment increased mean percentage of lumbar spine, while persistent loss of BMD at Lumbar spine was observed in the ZOL− group (Fig. 2b). Moreover, the mean longitudinal changes in lumbar spine t score (Fig. 2c) and z score (Fig. 2d) were notably increased in ZOL + group at different time points.

### ZOL treatment increased the expression of miR-302, miR-101 and miR-145 in the serum of HIV patients receiving tenofovir treatment

**Fig. 3:**
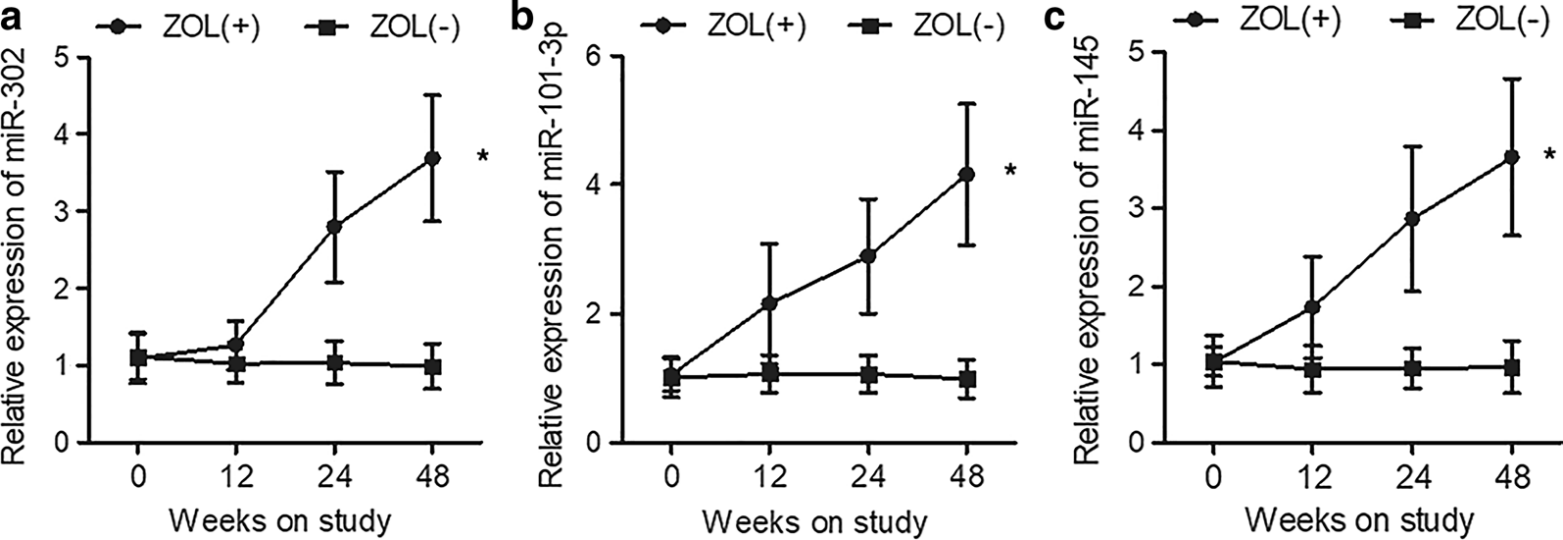
The expression of miR-302, miR-101 and miR-145 in the serum of HIV patients receiving tenofovir treatment was increased by ZOL treatment. **a** ZOL treatment enhanced the expression of miR-302 in the serum of HIV patients receiving tenofovir treatment. **b** ZOL treatment enhanced the expression of miR-101 in the serum of HIV patients receiving tenofovir treatment. **c** ZOL treatment enhanced the expression of miR-145 in the serum of HIV patients receiving tenofovir treatment

Peripheral blood samples were collected from patients in ZOL + and ZOL− groups at 0, 12, 24 and 48 weeks. Serum was isolated and qPCR was performed to analyze the expression of miR-302, miR-101 and miR-145 at different time points. As shown in Fig. 3, the expression of miR-302 (Fig. 3a), miR-101 (Fig. 3b) and miR-145 (Fig. 3c) in the serum of ZOL + patients was significantly higher than that in the serum of ZOL− patients at each time point. It is worth noting that the expression of miR-302, miR-101 and miR-145 in the serum of ZOL + patients was progressively elevated at 12, 24 and 48 weeks.

### ZOL treatment increased the expression of RANKL, SMAD3 and PRKACB in the serum of HIV patients receiving tenofovir treatment

**Fig. 4:**
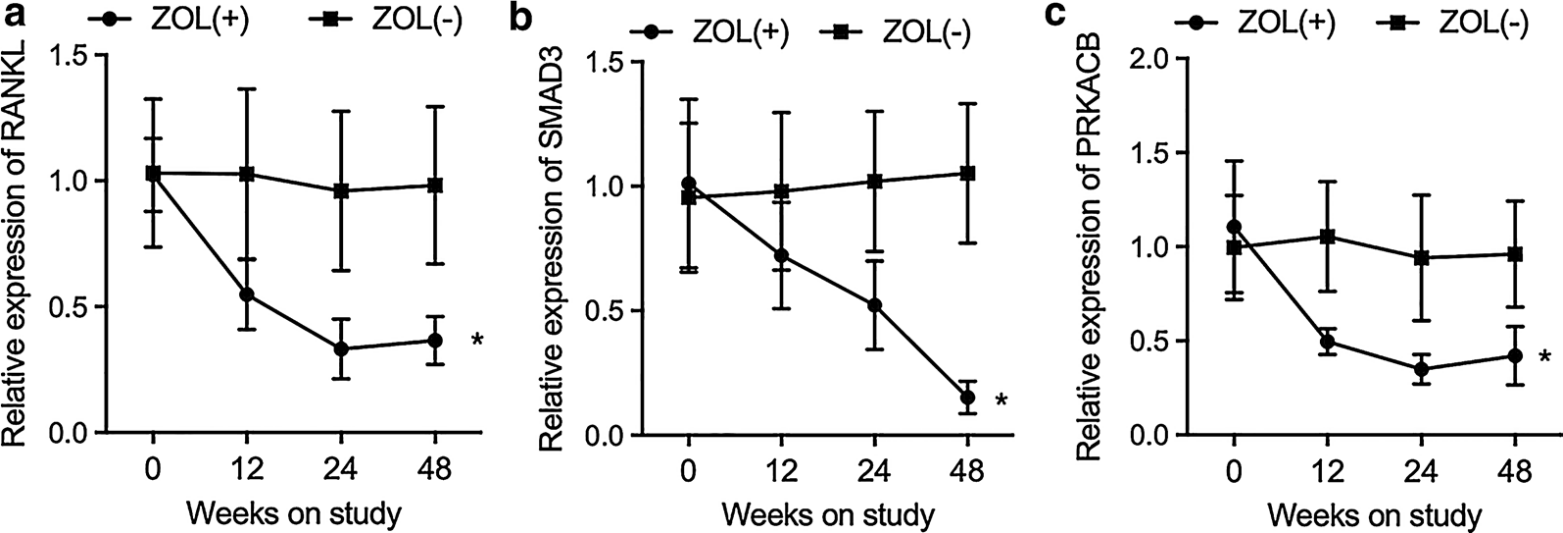
ELISA analysis showed that the expression of RANKL, SMAD3 and PRKACB was remarkably decreased in the serum of HIV patients receiving tenofovir treatment. **a** ZOL treatment suppressed the expression of RANKL in the serum of HIV patients receiving tenofovir treatment. **b** ZOL treatment suppressed the expression of SMAD3 in the serum of HIV patients receiving tenofovir treatment. **c** ZOL treatment suppressed the expression of PRKACB in the serum of HIV patients receiving tenofovir treatment

ELISA was performed to evaluate the expression of RANKL, SMAD3 and PRKACB in the serum of HIV patients receiving tenofovir treatment. The expression of RANKL (Fig. 4a), SMAD3 (Fig. 4b) and PRKACB (Fig. 4c) in patients receiving ZOL treatment was apparently decreased. The expression of RANKL, SMAD3 and PRKACB in the serum of ZOL + group gradually decreased at 0 week, 12 weeks, 24 weeks and 48 weeks.

### MiR-302, miR-101 and miR-145 suppressed the expression of PRKACB, RANKL and SMAD3 through binding to their 3′ UTR, respectively

**Fig. 5:**
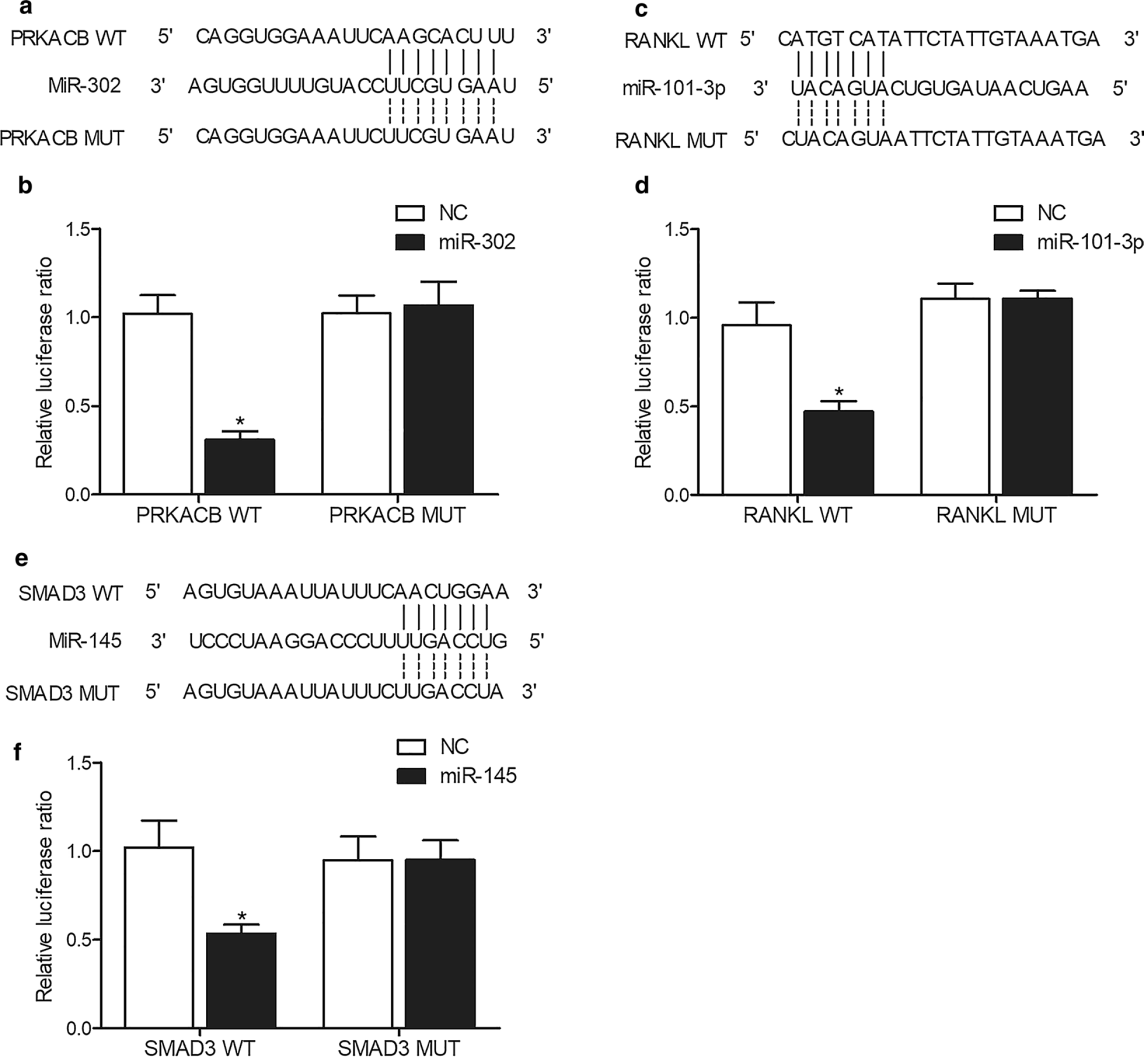
MiR-302, miR-101 and miR-145 suppressed the expression of PRKACB, RANKL and SMAD3 through binding to their 3′ UTR, respectively (*P value < 0.05 vs. NC group). **a** Sequence analysis indicated potential binding of miR-302 to the 3′ UTR of PRKACB. **b** The luciferase activity of wild type PRKACB vector was suppressed by miR-302 in human osteoclast precursor cells. **c** Sequence analysis indicated potential binding of miR-101 to the 3′ UTR of RANKL. **d** The luciferase activity of wild type RANKL vector was suppressed by miR-101 in human osteoclast precursor cells. **e** Sequence analysis indicated potential binding of miR-145 to the 3′ UTR of SMAD3. **f** The luciferase activity of wild type SMAD3 vector was suppressed by miR-145 in human osteoclast precursor cells

Binding target screening of miR-302, miR-101 and miR-145 indicated that miR-302 could bind to the 3′ UTR of PRKACB (Fig. 5a), miR-101 could bind to the 3′ UTR of RANKL (Fig. 5c) and miR-145 could bind to the 3′ UTR of SMAD3 (Fig. 5e). Luciferase vectors containing wild type and mutant PRKACB, RANKL and SMAD3 were established and transfected into human osteoclast precursor cells along with miR-302, miR-101 and miR-145, respectively. The luciferase activities of wild type PRKACB (Fig. 5b), RANKL (Fig. 5d) and SMAD3 (Fig. 5f) were effectively inhibited by miR-302, miR-101 and miR-145, respectively, whereas the luciferase activities of mutant vectors remained unchanged.

### ZOL inhibited RANKL‑induced expression of osteoclast‑specific genes

**Fig. 6:**
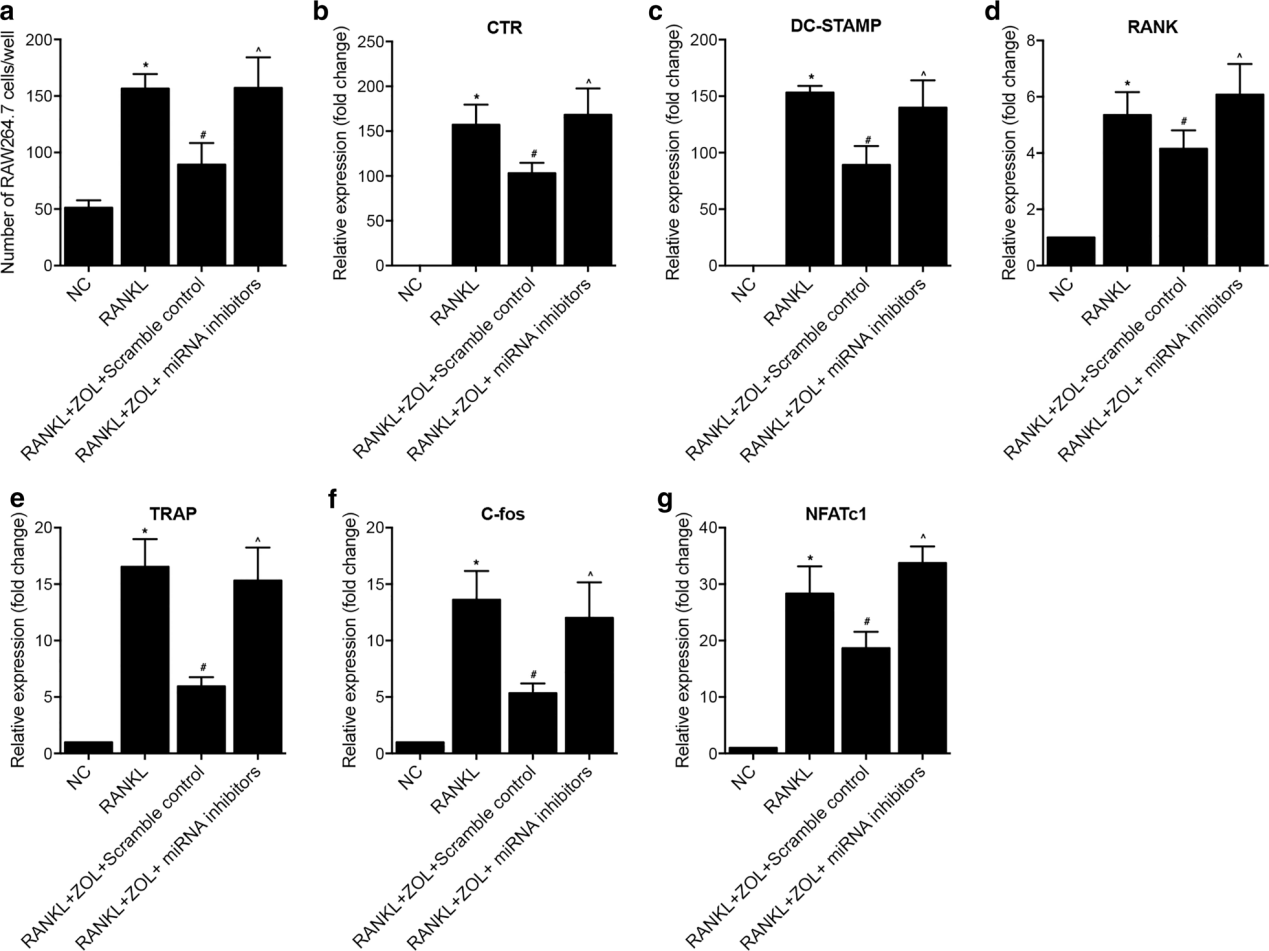
Quantitative real-time PCR indicated that the upregulation of the expression of osteoclast‑specific genes induced by RANKL was suppressed by ZOL treatment (*P value < 0.05 vs. NC group; #P value < 0.05 vs. RANKL group; ^P value < 0.05 vs. RANKL + ZOL + Scramble control group). **a** ZOL treatment inhibited RANKL-induced increase in the number of human osteoclast precursor cells, which was obstructed by the knockdown of miR-302, miR-101 and miR-145. **b** ZOL treatment inhibited RANKL-induced increase of RNA expression of CTR, which was obstructed by the knockdown of miR-302, miR-101 and miR-145. **c** ZOL treatment inhibited RANKL-induced increase of RNA expression of DC-STAMP, which was obstructed by the knockdown of miR-302, miR-101 and miR-145. **d** ZOL treatment inhibited RANKL-induced increase of RNA expression of RANK, which was obstructed by the knockdown of miR-302, miR-101 and miR-145. **e** ZOL treatment inhibited RANKL-induced increase of RNA expression of TRAP, which was obstructed by the knockdown of miR-302, miR-101 and miR-145. **f** ZOL treatment inhibited RANKL-induced increase of RNA expression of c-fos, which was obstructed by the knockdown of miR-302, miR-101 and miR-145. **g** ZOL treatment inhibited RANKL-induced increase of RNA expression of NFATc1, which was obstructed by the knockdown of miR-302, miR-101 and miR-145

In order to further explore the effect of ZOL treatment on the osteoclast formation and resorptive function, we treated human osteoclast precursor cells with RANKL and then ZOL. The number of human osteoclast precursor cells was dramatically increased by RANKL treatment, but ZOL treatment effectively reduced the number of human osteoclast precursor cells (Fig. 6a). Moreover, RANKL induced the upregulation of CTR (Fig. 6b), DC-STAMP (Fig. 6c), RANK (Fig. 6d), TRAP (Fig. 6e), c-FOS (Fig. 6f) and NFATc1 (Fig. 6g), while ZOL treatment obviously repressed the expression of above genes in the RANKL + ZOL + Scramble control group. Moreover, compared with the transfection with miRNA inhibitor scramble controls, the transfection of serval miRNA inhibitors including miR-302 inhibitors, miR-101 inhibitors and miR-145 inhibitors restored the reduced number of human osteoclast precursor cells (Fig. 6a) as well as the suppressed gene expressions (Fig. 6b–g).

### ZOL specifically attenuated RANKL‑induced activation of NF‑κB and JNK signaling pathways

**Fig. 7:**
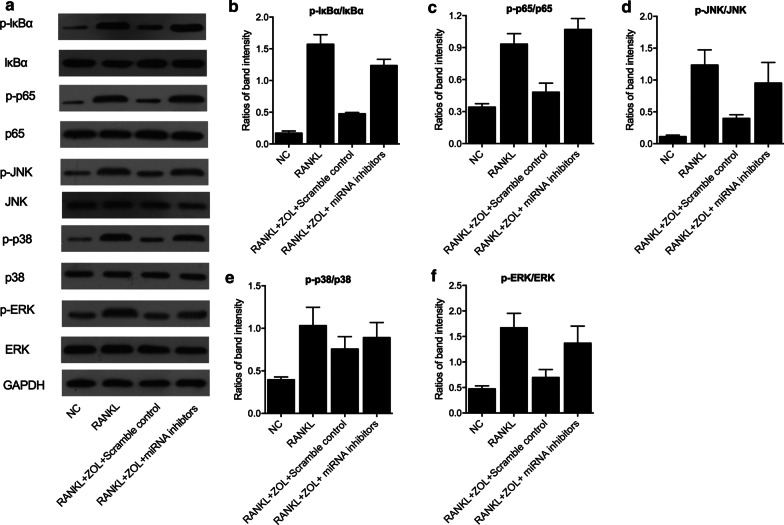
RANKL‑induced activation of NF‑κB and JNK signaling pathways was attenuated by ZOL treatment in human osteoclast precursor cells (*P value < 0.05 vs. NC group; #P value < 0.05 vs. RANKL group; ^P value < 0.05 vs. RANKL + ZOL + Scramble control group). **a** Western blot analysis showed ZOL treatment restored the normal expression of genes related to NF‑κB and JNK signaling pathways, while the knockdown of miR-302, miR-101 and miR-145 reversed this situation. **b** Quantitative analysis of Western blot showed that RANKL induced-upregulation of p-lκBα/lκBα expression was suppressed by ZOL treatment, while the knockdown of miR-302, miR-101 and miR-145 reversed this situation. **c** Quantitative analysis of Western blot showed that RANKL induced-upregulation of p-p65/p65 expression was suppressed by ZOL treatment, while the knockdown of miR-302, miR-101 and miR-145 reversed this situation. **d** Quantitative analysis of Western blot showed that RANKL induced-upregulation of p-JNK/JNK expression was suppressed by ZOL treatment, while the knockdown of miR-302, miR-101 and miR-145 reversed this situation. **e** Quantitative analysis of Western blot showed that RANKL induced-upregulation of p-p38/p38 expression was suppressed by ZOL treatment, while the knockdown of miR-302, miR-101 and miR-145 reversed this situation. **f** Quantitative analysis of Western blot showed that RANKL induced-upregulation of p-ERK/ERK expression was suppressed by ZOL treatment, while the knockdown of miR-302, miR-101 and miR-145 reversed this situation

Western blot was performed to assess the effect of ZOL treatment on the NF-kB and JNK pathways (Fig. 7a). RANKL treatment apparently enhanced the expression of p-lκBα/lκBα (Fig. 7b), p-p65/p65 (Fig. 7c), p-JNK/JNK (Fig. 7d), p-p38/p38 (Fig. 7e) and p-ERK/ERK (Fig. 7f) in human osteoclast precursor cells. ZOL treatment obviously suppressed RANKL-induced activation of p-lκBα/lκBα (Fig. 7b), p-p65/p65 (Fig. 7c), p-JNK/JNK (Fig. 7d), p-p38/p38 (Fig. 7e) and p-ERK/ERK (Fig. 7f) expression in human osteoclast precursor cells. Moreover, the knockdown of miR-302, miR-101 and miR-145 by miRNA inhibitors partly restored the reduced protein parameters (Fig. 7b–f).

## Discussion

HIV-infected individuals show osteopenia/osteoporosis as well as bone loss (Crignis et al. 2008; El-Maouche et al. 2011; Fessel et al. 2011). Furthermore, osteopenia development as well as the enhancement of osteoporosis is related to anti-retroviral therapy (Malizia et al. 2007; Grigsby et al. 2010; Briot et al. 2011). Considering that osteoclasts are responsible for bone resorption and are the key mediator of constant bone loss, the recognition of factors enhancing or preventing osteoclastic activities will certainly assist in developing reliable methods to treat osteoporosis caused by HIV as well as cancer. In fact, it was shown that monocyte derived macrophages (MDM) in HIV-infected subjects are morphologically as well as functionally associated with osteoclasts by showing higher affinity to RANKL. Furthermore, it was revealed that hemin hinder osteoclast development induced by HIV, obstructing RANKL activity to avoid osteoclastogenesis (Takeda et al. 2015).

ZOL is an appealing option for the therapy of osteoporosis in HIV-infected people due to its excellent tolerability as well as the dosing method of intravenous infusion. It was observed that ZOL was well endured, without any negative and severe effects, although appropriate hydration is suggested to minimize the probability of flu-like signs. Nonetheless, serious ZOL-induced issues, including uveitis, have actually been reported (Bolland et al. 2012). In a multicenter study, a 3-year treatment with ZOL minimized the danger of vertebral fracture by over 70% (Jacques et al. 2012). Likewise, in a ZOL study, the 2-year treatment with this medicine minimized the occurrence of vertebral fracture by over 60% (Taguchi et al. 2019). In this study, we enrolled HIV patients receiving tenofovir treatment who were diagnosed with osteoporosis to evaluate the effect of ZOL, which suppressed CTx and Osteocalcin expression and prevented BMD loss. In addition, we isolated the serum from ZOL treated and untreated HIV patients receiving tenofovir treatment, and performed qPCR to analyze the expression of miR-302, miR-101 and miR-145. Significant elevation of miR-302, miR-101 and miR-145 expression was observed in patients receiving ZOL treatment.

In a microarray study of > 300 miRNAs, the treatment of breast cancer with a low-dose therapy of ZOL considerably altered the expression of > 50 miRNAs. 9 upregulated as well as 12 down-regulated miRNAs were identified after a 24 h therapy. Additionally, ZOL boosted the expression of 10 miRNAs, consisting of miR-145, miR-101-1, miR-139, miR-129, as well as miR-124-1, while decreasing the expression of 20 miRNAs (Fanale et al. 2016b). It was additionally revealed that miR-145 boosted the differentiation to osteoblasts while preventing RANKL-induced osteoclastogenesis (Yu et al. 2018; Sun et al. 2016). Nonetheless, miR-145 silencing rescued femoral head necrosis (Tian et al. 2017). An increase in the expression of miR-21 and miR-23a, as well as a decrease in the expression of miR-145, was found in BRONJ. Nevertheless, the relationship of intracellular miR expression to circulating miR expression is unidentified (Yang et al. 2018).

Bone is continuously renewed via osteoclastic resorption and osteoblastic formation of the bone. The RANKL-RANK pathway is necessary for human osteoclastogenesis. The loss of RANK or RANKL triggers osteopetrosis as a result of the absence of osteoclasts (Kong et al. 1999; Takayanagi 2007). Regardless of data showing the presence of osteoclastogenesis independent of RANKL, many studies do not have adequate proof to confirm RANKL independence (Hemingway et al. 2013, 2011). PRKACB was predicted to be targeted by miR-302a-3p in human mandibular osteoblast-like cells (Irwandi et al. 2018). Since miR-302a-3p could also suppress expression of RANKL expression in HMOBs within PGE2-IFNγ regulatory network, PRKACB was also involved in this network (Irwandi et al. 2018). Also, by regulating SMAD3 and its downstream target genes, miR-300 was proved to negatively regulate the differentiation of osteoblasts in the management of bone-related disorder management strategies (Kaur et al. 2020). In this study, we found that ZOL treatment effectively enhanced the expression of RANKL, SMAD3 and PRKACB. Furthermore, the luciferase activities of wild type PRKACB, RANKL and SMAD3 were significantly repressed by miR-302, miR-101 and miR-145, respectively.

Experiments making use of HIV transgenic rats showed decreased BMD, increased bone resorption, as well as reduced BMI upon boosted synthesis of RANKL from B cells (Vikulina et al. 2010). According to the data, the HIV-infected subjects undergoing no treatment showed a correlation between the proportion of RANKL/OPG and the BMD of hip, which is most likely triggered by the imbalances of RANKL/OPG (Titanji et al. 2014; Moran et al. 2017). SMAD3 is crucial in the transduction of osteoclastogenic signal of RANKL/RANK. The important function of SMAD3 in RANKL-induced osteoclastogenesis is even more obvious when SMAD3 expression is silenced by utilizing shSMAD3 (Yasui et al. 2011).

## Conclusion

The findings of this study demonstrated that the administration of ZOL suppressed the expression of RANKL via modulating signaling pathways of miR-101-3p/RANKL, miR-302/PRKACB/RANKL and miR-145/SMAD3/RANKL. Furthermore, ZOL treatment alleviated osteoporosis in HIV subjects on tenofovir treatment.

## Data Availability

The datasets used and/or analyzed during the current study are available from the corresponding author on reasonable request.

## Abbreviations

ZOL: Zoledronate
RANKL: Receptor activator of nuclear-κB ligand
